# Changes in the tumor microenvironment in recurrent head and neck squamous cell carcinoma and its implication on efficacy of immune checkpoint inhibitors

**DOI:** 10.1007/s12672-024-01504-0

**Published:** 2024-11-20

**Authors:** Dong Hyun Kim, Mingu Kang, Gahee Park, Mohammad Mostafavi, Yoojoo Lim, Chan-Young Ock, Jiwon Koh, Yoon Kyung Jeon, Kyeong Cheon Jung, Soon-Hyun Ahn, Eun-Jae Chung, Seong-Keun Kwon, Bhumsuk Keam

**Affiliations:** 1 Department of Internal Medicine, Seoul National University Hospital, 101, Daehak-Ro, Jongro-Gu, Seoul, 03080 Republic of Korea; 2 Department of Translational Medicine, Seoul National University College of Medicine, Seoul, Republic of Korea; 3 Lunit, Seoul, Republic of Korea; 4 Department of Pathology, Seoul National University Hospital, Seoul, Republic of Korea; 5 Cancer Research Institute, Seoul National University College of Medicine, Seoul, Republic of Korea; 6 Department of Otorhinolaryngology-Head and Neck Surgery, Seoul National University Hospital, Seoul, Republic of Korea

## Abstract

Little is known about changes in the abundance of tumor-infiltrating lymphocytes (TILs) and immune phenotype (IP) in recurrent and/or metastatic (R/M) head and neck squamous cell carcinoma (HNSCC). We aimed to compare the TILs and IP between initial and recurrent HNSCCs using paired analysis. Thirty-seven patients who experienced recurrence after surgical resection and received treatment with immune checkpoint inhibitors (ICIs) between June 2014 and June 2023 were included. Changes in intratumoral TIL (iTILs), stromal TIL (sTILs), and IPs were subjected to paired analysis between the initial and recurrent tumors. We investigated their relationship with the outcomes of ICIs. The density of iTIL and sTIL in the recurrent tumors was significantly lower compared to initial tumors. IP was significantly different; the proportion of desert IP was higher in recurrent tumors (83.8% vs. 35.1%, *P* < 0.001). Increased sTIL was a favorable indicator for overall response to ICIs and progression-free survival. Our findings suggest TILs decrease during recurrence compared with the initial tumor, resulting in a transition toward desert IP. Therefore, careful evaluation of TIL density in both initial and recurrent tumors is recommended when using ICIs in patients with R/M HNSCC.

**Supplementary Information:**

The online version contains supplementary material available at 10.1007/s12672-024-01504-0.

## Introduction

Approximately 50% of the patients with locally advanced head and neck squamous cell carcinoma (HNSCC) experience recurrence after radical surgery or concurrent chemoradiotherapy. Palliative chemotherapy is administered for recurrent and/or metastatic (R/M) HNSCC; however, the prognosis of these patients remains unfavorable [1]. Immune checkpoint inhibitors (ICIs) may be a viable option; however, the objective response rates remain low. Therefore, it is crucial to find predictive biomarkers [2–6].

Factors in the tumor microenvironment (TME) such as programmed death-ligand 1 (PD-L1) positivity and tumor-infiltrating lymphocytes (TIL) can serve as biomarkers to predict the efficacy of ICIs in HNSCC [7, 8]. TILs are directly related to anticancer immunity and significantly affect the efficacy of ICIs [9, 10]. However, there is no consensus on using initial or recurrent tumor tissues. Although the TME may change after tumor progression, whether the initial TME still has predictive value when using ICI after recurrence is unclear. Moreover, there are differences in findings on TME changes upon recurrence compared to the initial state [11–13]. Therefore, conducting paired comparisons of the TME between recurrence and initial presentation in HNSCC and analyzing their relevance to the efficacy of ICI is expected to provide valuable insights into the immune biomarkers associated with ICI usage in R/M HNSCC.

TIL and immune phenotype (IP) analyzed using Lunit SCOPE IO, an artificial intelligence (AI)-powered TIL analyzer, have predictive value for using ICI in multiple cancer types [14, 15]. Therefore, we analyzed TIL density and IP using the Lunit SCOPE IO in both initial and recurrent HNSCC. The aim of this study was to compare the TME of initial tumor tissue and recurrent tumor tissue of HNSCC using paired analysis, and to evaluate the predictive value of the initial and recurrent TME when using ICIs.

## Material and methods

### Patients and study design

Patients with histologically confirmed, R/M HNSCC who were treated with ICIs between June 2014 and June 2023 were included. Patients who had undergone surgical resection for initial HNSCC but later experienced locoregional recurrence or distant metastasis and subsequently received ICIs were eligible for the study. Patients with nasopharyngeal carcinoma and those with distant metastases at initial diagnosis were excluded. Only patients with hematoxylin and eosin (H&E)-stained archival tumor tissues from both the initial tumor and recurrence for paired analysis of TIL and IP using Lunit SCOPE IO were included in the study. Finally, 37 consecutive patients were included in the final analysis. Three pathologists (JK, YKJ, and KCJ) reviewed all the histopathological slides to confirm the diagnosis.

Medical records were reviewed for baseline characteristics, ICI type, ICI response, and survival outcomes. Human papillomavirus testing was conducted using p16 immunohistochemistry (clone E6H4, Ventana Medical Systems, Tucson, AZ, USA). Tumor response was assessed using RECIST version 1.1. Progression-free survival (PFS) was defined as the time from ICI administration to disease progression or death of any cause. Overall survival (OS) was defined as the time from ICI administration to death from any cause.

### Procedures

The Lunit SCOPE IO (Lunit Inc., Seoul, Republic of Korea) is an AI-powered spatial TIL analyzer that identifies and quantifies TILs within the cancer epithelium (intratumoral TIL; iTIL) and stroma (stromal TIL; sTIL) from H&E-stained whole-slide images (WSIs). It comprises two convolutional neural networks, one of which segments the cancer area (CA) and cancer-related stroma (CS), whereas the other identifies TILs. The Lunit SCOPE IO was originally trained and optimized using 2.8 × 10^9^ μm^2^ H&E-stained tissue regions containing 6.0 × 10^5^ TILs, extracted from 3,166 WSI assorted from 25 different tumor types, and annotated by board-certified pathologists [15, 16]. The model used in this study was updated by training and optimization using 1.4 × 10^10^ μm^2^ of CA and CS, including 6.23 × 10^5^ TILs, extracted from 18,679 H&E-stained WSI of 17 different solid tumor types, including HNSCC.

The detailed process of TIL analysis using Lunit SCOPE IO has been described in our previous study [17, 18]. Specifically, the model segmented WSIs into CA and CS, and identified and quantified the TILs in each area. The model estimated the density of iTILs or sTILs per 1 mm^2^ of the corresponding tissue area for each case. The model used iTIL and sTIL densities in 0.25 mm^2^-sized grids to derive IP of each grid; that is (i.e.), inflamed-grids having iTIL density of ≥ 130/mm^2^; immune-excluded-grids having sTIL density of ≥ 260/mm^2^ and iTIL density of < 130/mm^2^ and immune-desert, i.e., grids having iTIL and sTIL densities of < 130/mm^2^ and < 260/mm^2^, respectively. The inflamed score (IS), immune-excluded score (IES), and immune-desert score (IDS) of the WSIs were defined as the number of grids annotated to a certain IP divided by the total number of grids analyzed in the WSI. Finally, the representative IP for each WSI was defined as inflamed IP if the IS was ≥ 20.0%, as immune-excluded IP if the IES was ≥ 33.3% and IS was < 20.0%, else it was considered an immune-desert IP. TIL and immune score thresholds to determine the grid- and WSI-level IP classification were determined before the study as the value that optimally predicted high interferon-γ–responsive gene signature levels in a set of TCGA pan-carcinoma tumor samples (N = 7454) [19, 20].

### Statistical analysis

Descriptive statistics were presented as median values with ranges or numbers with percentiles. Wilcoxon signed-rank tests were used for paired data, and Spearman’s rank tests were used to assess the correlations between the two variables. For unpaired data, categorical variables between the two groups were compared using Fisher’s exact test or the chi-square test, and *P* values were two-sided. Differences in the means or medians of the continuous variables between the two groups were assessed using the Wilcoxon rank-sum test. The cut-off for discriminating between low and high IP score levels was defined as the point with the highest log-rank statistical level for PFS by the maximally selected rank statistics for each biomarker. The Kaplan–Meier method was used to determine the PFS and OS. The log-rank test was used to assess differences between the groups in PFS and OS. We performed a backward-selection multivariate logistic regression analysis to identify the relevant factors for the overall response, and estimates were provided with odds ratio (ORs) and 95% confidence intervals (CIs). Factors associated with PFS and OS were evaluated using the Cox proportional hazards model, and hazard ratios (HRs) with 95% CIs were calculated. The statistical software ‘R’ version 4.1.3 (www.r-project.org) was used for all statistical analyses. *P* values < 0.05 were considered statistically as significant.

## Results

### Patient and disease characteristics

The baseline characteristics of the 37 patients are summarized in Table 1. The median patient age was 57 years (range, 32–82), and 62.2% were males. The primary tumor was located in the oral cavity in 45.9% of the patients, and 64.9% were in initial stage IV. A total of 29 patients received post-surgical adjuvant treatment; 15 underwent concurrent chemoradiotherapy, 11 received radiotherapy, and 3 received chemotherapy. Most (73%) patients were treated with PD-1 inhibitors.

**Table 1:** Baseline characteristics

	N = 37
Median age, years (range)	57 (32–82)
Age > 60, n (%)	15 (40.5)
Sex, n (%)	
Male	23 (62.2)
Female	14 (37.8)
Current or former smoker, n (%)	12 (32.4)
Primary tumor location, n (%)	
Oral cavity	17 (45.9)
Oropharynx	15 (40.5)
Hypopharynx	2 (5.4)
Nasal cavity	3 (8.1)
p16 status for oropharyngeal carcinoma, n (%)	*N = 15*
Positive	9 (60.0)
Negative	6 (40.0)
Initial stage, n (%)	
I–III	13 (35.1)
IV	24 (64.9)
Number of previous lines of chemotherapy, n (%)	
0	7 (18.9)
1	10 (27.0)
2 or more	20 (54.1)
Immune checkpoint inhibitor treatment, n (%)	
PD-1 inhibitor	27 (73.0)
Pembrolizumab	15 (40.5)
Nivolumab	12 (32.4)
PD-L1 inhibitor	5 (13.5)
Durvalumab	4 (10.8)
Avelumab	1 (2.7)
PD-1/PD-L1 inhibitor + CTLA-4 inhibitor	5 (13.5)
Nivolumab + Ipilimumab	2 (5.4)
Durvalumab + Tremelimumab	3 (8.1)
Time between initial tissue acquisition and ICI use, day, median (range)	476 (70–1789)
Time between recurrent tissue acquisition and ICI use, day, median (range)	27 (6–1353)
Time to recurrence, day, median (range)	323 (47–1609)
< 1 year, n (%)	20 (54.1)
< 2 years, n (%)	29 (78.4)
< 3 years, n (%)	32 (86.5)

*PD-1* programmed cell death-1, *PD-L1* programmed cell death-ligand 1, *CTLA-4* cytotoxic T-lymphocyte-associated protein 4, *ICI* immune checkpoint inhibitor

Fourteen (37.8%) patients underwent a second biopsy for recurrence at the primary site, 11 (29.7%) at cervical lymph node metastasis, and 12 (32.4%) at distant metastasis sites (Supplementary Table 1). The median durations from initial tissue collection through surgery to ICI use and from tissue collection at recurrence to ICI use were 476 and 27 days, respectively. The median duration between the initial and recurrent occurrence was 323 days (range, 47–1609).

### Comparison of tumor microenvironment between initial and recurrent tumors

The results of the analysis using the Lunit SCOPE IO are presented in Table 2. In initial and recurrent tumors, the median iTIL density was 57.71/mm^2^ (range, 0.55–753.19) and 35.41/mm^2^ (range, 1.94–145.48), while the median sTIL density was 753.55/mm^2^ (range, 25.34–8725.75) and 214.85/mm^2^ (range, 3.13–1523.06), respectively. The IP of each 1mm^2^ grid was determined for spatial analysis of TIL distribution. In the initial tumors, the median IS, IES and IDS values were 8.33%, 29.77%, and 45.89%, respectively, whereas in recurrent tumors, these were 5.05%, 14.29%, and 74.75%, respectively.

**Table 2:** Comparison of tumor microenvironment between initial and recurrent tumor

	Initial tumor N = 37	Recurrence N = 37	*P*-value
Median TIL density, /mm^2^ (range)			
Intratumoral	57.71 (0.55–753.19)	35.41 (1.94–145.48)	0.010
Stromal	753.55 (25.34–8725.75)	214.85 (3.13–1523.06)	< 0.001
Immune phenotype score, %, median (range)			
Inflamed score	8.33 (0–73.95)	5.05 (0–33.7)	0.007
Immune-excluded score	29.77 (0–80.28)	14.29 (0–71.91)	0.001
Immune desert score	45.89 (0.84–100)	74.75 (22.36–100)	< 0.001
Immune phenotype, n (%)			< 0.001
Inflamed	11 (29.7)	1 (2.7)	
Immune-excluded	13 (35.1)	5 (13.5)	
Immune desert	13 (35.1)	31 (83.8)	

*TIL* tumor-infiltrating lymphocyte

**Fig. 1:**
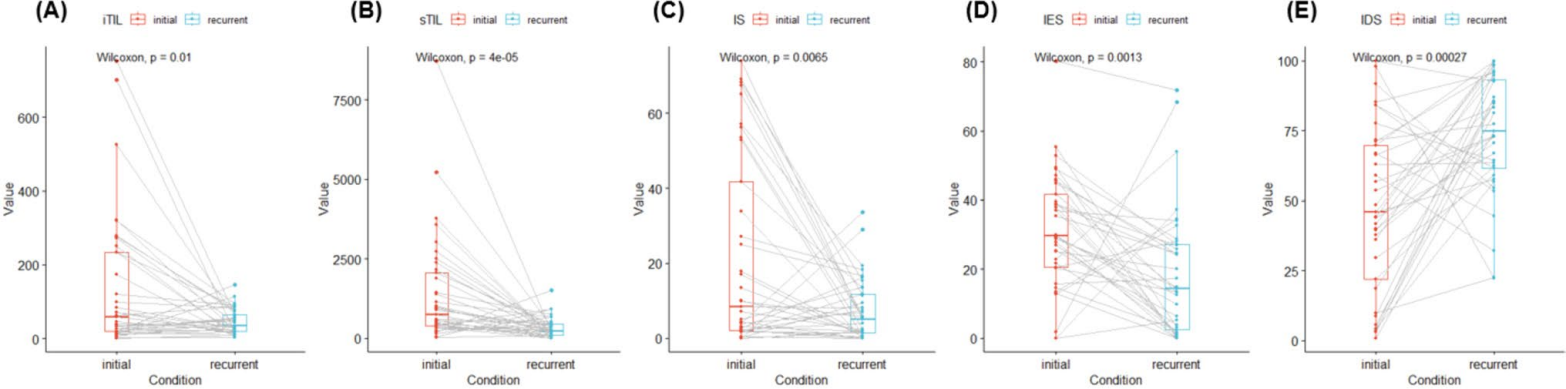
Changes in tumor microenvironment between the primary and recurrent tumor. **A** Changes in the density of intratumoral tumor-infiltrating lymphocyte; **B** Changes in the density of stromal tumor-infiltrating lymphocyte; **C** Changes in the inflamed score; **D** Changes in the immune-exclude score; **E** Changes in the immune-desert score

Both iTIL and sTIL levels were significantly lower in the recurrence group than in the initial tumor group (Fig. 1). Significantly lower IS and IES were observed in recurrence than in the initial tumor, whereas IDS was higher in recurrent tumors. When examining the correlation between iTIL, sTIL, IS, IES, and IDS in the initial tumor and recurrence, some correlations were observed. However, there were no associations between the factors in the initial tumor and those associated with recurrent tumor (Supplementary Fig. 1).

**Fig. 2:**
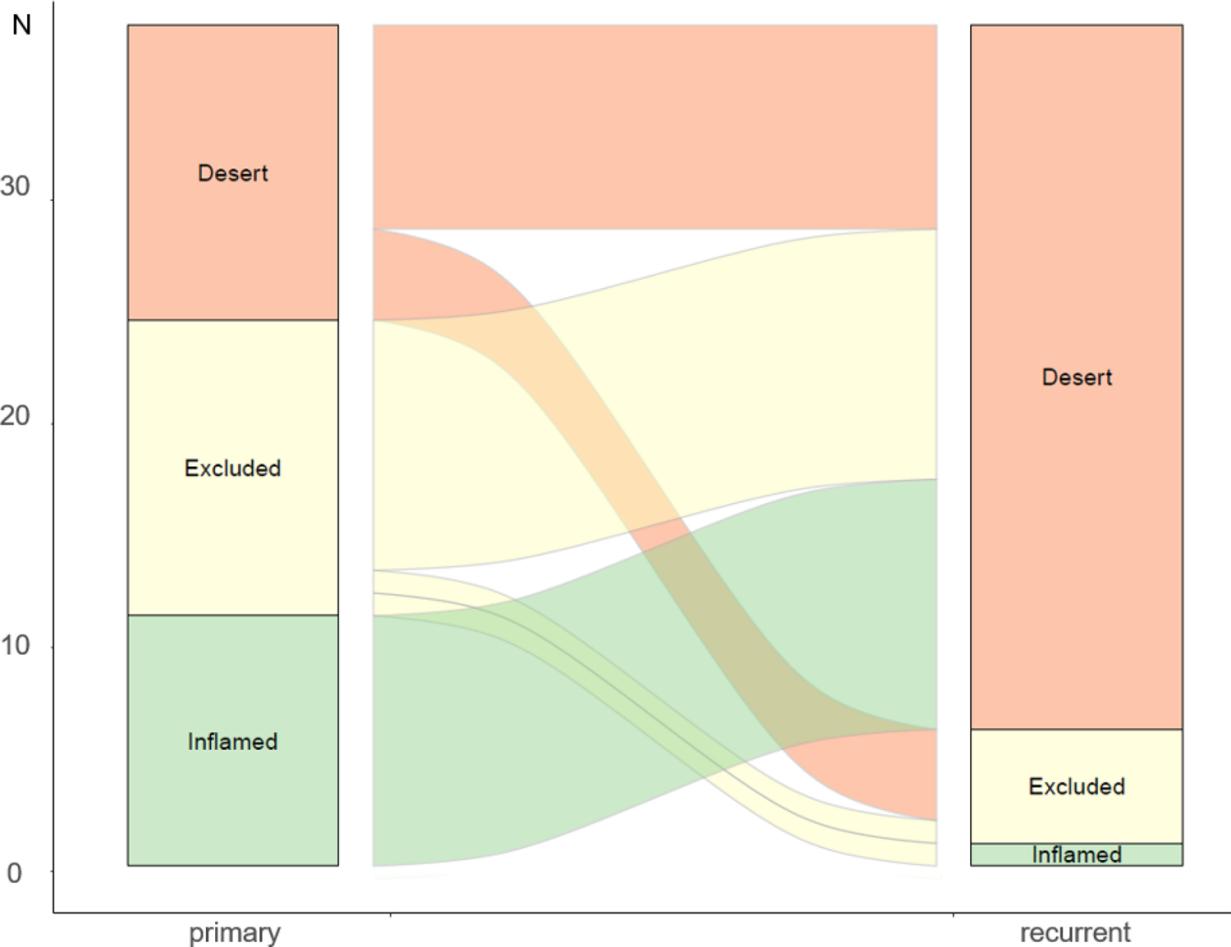
Flow chart (Sankey plot) showing changes in the immune phenotype

There was a significant difference in the IP between the initial tumor and recurrence (*P* < 0.001). Regarding recurrence, most IPs were classified as immune-deserts, with a higher proportion (83.8%) than 35.1% in the initial tumor. Notably, all the inflamed and most of the immune-excluded IP observed in the initial tumor turned into an immune-desert IP during recurrence (Fig. 2).

### Changes in the tumor-infiltrating lymphocytes in recurrent tumors

We classified patients into two groups to investigate factors affecting changes in TIL density. Patients whose TILs increased during recurrence compared to the initial tumor were defined as ‘increased’ group, while those with decreased TILs were categorized as the ‘decreased’ group. Based on iTILs, 23 patients (62.2%) were included in the decreased group, whereas based on sTILs, 30 patients (81.1%) belonged to the decreased group. The comparisons between the two groups are presented in Table 3. The decreased iTIL group was younger than the increased iTIL group (median, 55 vs. 66 years, *P* = 0.003). The proportion of patients with decreased sTIL levels was higher in the decreased iTIL group (95.7 vs. 57.1%, *P* = 0.014). There were no significant differences in baseline characteristics between the two groups based on sTIL levels. No significant relationship was observed between the time interval between tissue collection and changes in the TIL density.

**Table 3:** Comparison between patients with increased TIL and those with decreased TIL

	Intratumoral TIL			Stromal TIL		
	Increased	Decreased	*P*-value	Increased	Decreased	*P*-value
	(N = 14)	(N = 23)		(N = 7)	(N = 30)	
Median age, years (range)	66 (50–82)	55 (32–78)	0.003	62 (48–82)	56 (32–79)	0.269
Age > 60, n (%)	9 (64.3)	6 (26.1)	0.051	4 (57.1)	11 (36.7)	0.571
Sex, n (%)			0.209			0.898
Male	11 (78.6)	12 (52.2)		5 (71.4)	18 (60.0)	
Female	3 (21.4)	11 (47.8)		2 (28.6)	12 (40.0)	
Current or former smoker, n (%)	5 (35.7)	7 (30.4)	1.000	3 (42.9)	9 (30.0)	0.837
Primary tumor location, n (%)			0.948			0.825
Oral cavity	7 (50.0)	10 (43.5)		3 (42.9)	14 (46.7)	
Oropharynx	5 (35.7)	10 (43.5)		3 (42.9)	12 (40.0)	
Hypopharynx	1 (7.1)	1 (4.3)		0 (0)	2 (6.7)	
Nasal cavity	1 (7.1)	2 (8.7)		1 (14.3)	2 (6.7)	
Initial stage 4, n (%)	9 (64.3)	15 (65.2)	1.000	5 (71.4)	19 (63.3)	1.000
Differentiation, n (%)			0.331			0.206
Well	4 (28.6)	4 (17.4)		0 (0)	8 (26.7)	
Moderate	6 (42.9)	7 (30.4)		3 (42.9)	10 (33.3)	
Poorly	2 (14.3)	10 (43.5)		2 (28.6)	10 (33.3)	
Not assessed	2 (14.3)	2 (8.7)		2 (28.6)	2 (6.7)	
Time to recurrence, day, median (range)	333 (126–1194)	323 (47–1609)	0.672	297 (63–499)	330 (47–1609)	0.628
< 1 year, n (%)	7 (50.0)	13 (56.5)	0.963	4 (57.1)	16 (53.3)	1.000
< 2 years, n (%)	12 (85.7)	17 (73.9)	0.664	7 (100)	22 (73.3)	0.301
< 3 years, n (%)	13 (92.9)	19 (82.6)	0.698	7 (100)	25 (83.3)	0.584
Stromal TIL density, n (%)			0.014			
Increased	6 (42.9)	1 (4.3)				
Decreased	8 (57.1)	22 (95.7)				

TIL, tumor-infiltrating lymphocyte

### Clinical outcomes using immune checkpoint inhibitor based on changes in TILs

The response to ICIs was evaluated in 37 patients. As shown in Table 4, the best response to ICIs was complete response in six patients (16.2%), partial response in six patients (16.2%), stable disease in six patients (16.2%), and progressive disease in 19 patients (51.4%). The overall response rate was 32.4% (95% CI, 18.0%–49.8%). After a median follow-up of 10.1 months, the median PFS was 1.9 months (95% CI, 1.5–7.5 months), and the median OS was 14.2 months (95% CI, 8.3–31.1 months).

**Table 4:** Clinical outcomes of immune checkpoint inhibitor

	All N = 37	Increased sTIL N = 7	Decreased sTIL N = 30	*P*-value
Best response, n (%)				0.074
Complete response	6 (16.2)	2 (28.6)	4 (13.3)	
Partial response	6 (16.2)	3 (42.9)	3 (10.0)	
Stable disease	6 (16.2)	1 (14.3)	5 (16.7)	
Progressive disease	19 (51.4)	1 (14.3)	18 (60.0)	
Overall response rate, % (95% CI)	32.4 (18–49.8)	71.4 (29–96.3)	23.3 (9.9–42.3)	0.046
Disease control rate, % (95% CI)	48.6 (31.9–65.6)	85.7 (42.1–99.6)	36.7 (19.9–56.1)	0.054
PFS, months, median (95% CI)	1.9 (1.5–7.5)	NR (7.5-NR)	1.7 (1.3–5.2)	0.001
3 months PFS rate, % (95% CI)	48.7 (34.9–67.7)			
6 months PFS rate, % (95% CI)	37.6 (24.8–57.1)			
12 months FPS rate, % (95% CI)	26.1 (15–45.3)			
OS, months, median (95% CI)	14.2 (8.3–31.1)	NR (12.9-NR)	10.1 (7.2–30.7)	0.034
12 months OS rate, % (95% CI)	56.3 (41.8–75.9)			
24 months OS rate, % (95% CI)	37.3 (23.3–59.9)			
36 months OS rate, % (95% CI)	12.4 (3.9–39.7)			

*sTIL* stromal tumor-infiltrating lymphocyte, *CI* confidence interval, *PFS* progression-free survival, *OS* overall survival, *NR* not reached

We categorized patients into responders (complete response or partial response) and non-responders (stable disease or progressive disease) to identify factors related to response to ICIs. The IP of both the initial and recurrent tumor, and changes in iTIL density, were not associated with the overall response. In contrast, patients with decreased sTIL density showed a significantly lower overall response rate than those with increased sTIL density (23.3% vs. 71.4%, *P* = 0.046). In univariate analysis, age > 60 years and increased sTIL density were predictors of the overall response (Supplementary Table 2). Moreover, multivariate analysis showed that only increased sTIL density (OR 7.9, 95% CI 1.09–57.25, *P* = 0.041) was independent predictor of overall response (Table 5).

**Table 5:** Factors related to overall response and survival outcomes

Outcomes	Estimate (95% CI)	*P*-value
*Overall response rate*	Odds ratio	
Age (> 60 vs ≤ 60)	4.97 (0.99–24.9)	0.051
Changes in sTIL (increased vs decreased)	7.9 (1.09–57.25)	0.041
*Progression-free survival*	Hazard ratio	
Primary tumor site (oral vs others)	1.93 (0.93–4)	0.077
Changes in sTIL (increased vs decreased)	0.12 (0.03–0.53)	0.005
*Overall survival)*	Hazard ratio	
Changes in sTIL (increased vs decreased	0.24 (0.06–1.01)	0.052

*CI* confidence interval, *sTIL* stromal tumor-infiltrating lymphocyte

**Fig. 3:**
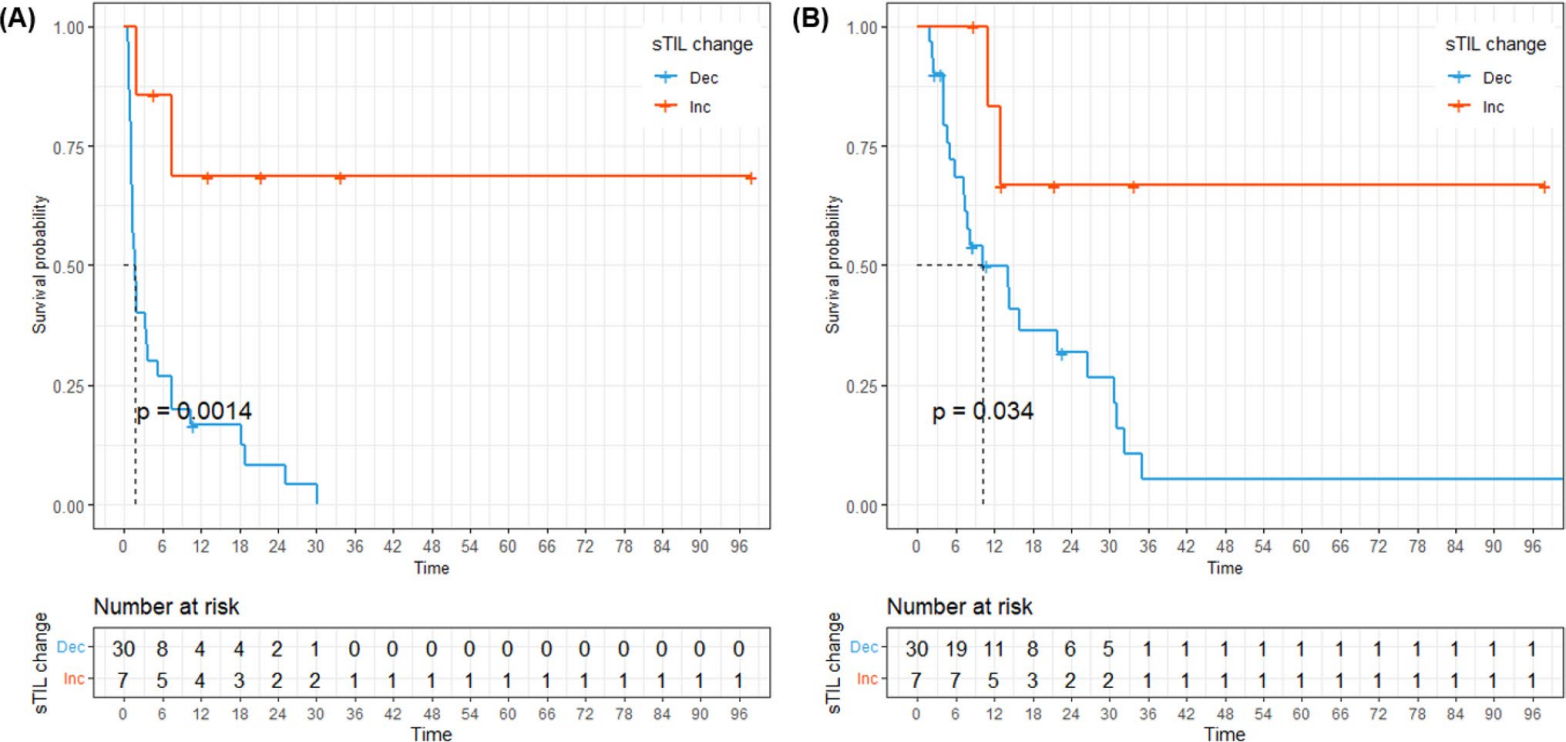
Survival outcomes based on changes in stromal tumor-infiltrating lymphocytes (TIL) density. **A** Progression-free survival; **B** Overall survival

We compared the survival outcomes between patients with increased and decreased TIL density. There was no difference in PFS (median 5.5 vs. 1.7 months, *P* = 0.18) and OS (median 10.9 vs. 16 months, *P* = 0.84) between increased and decreased iTIL group (Supplementary Fig. 2). On the contrary, patients whose recurrent tumors had increased sTIL showed both longer PFS (Fig. 3A, median NR vs. 1.7 months, *P* = 0.001) and OS (Fig. 3B, median NR vs. 10.1 months, *P* = 0.034). Supplementary Table 3 presents the outcomes of the univariate analysis for each parameter of PFS and OS. In univariate analysis, well differentiation and increased sTIL density were significant factors associated with PFS. The multivariate Cox proportional-hazards model showed that increased sTIL density (HR 0.12, 95% CI 0.03–0.53, *P* = 0.005) was the sole significant indicator of PFS.

### Immune phenotype score and clinical outcomes of immune checkpoint inhibitor

**Fig. 4:**
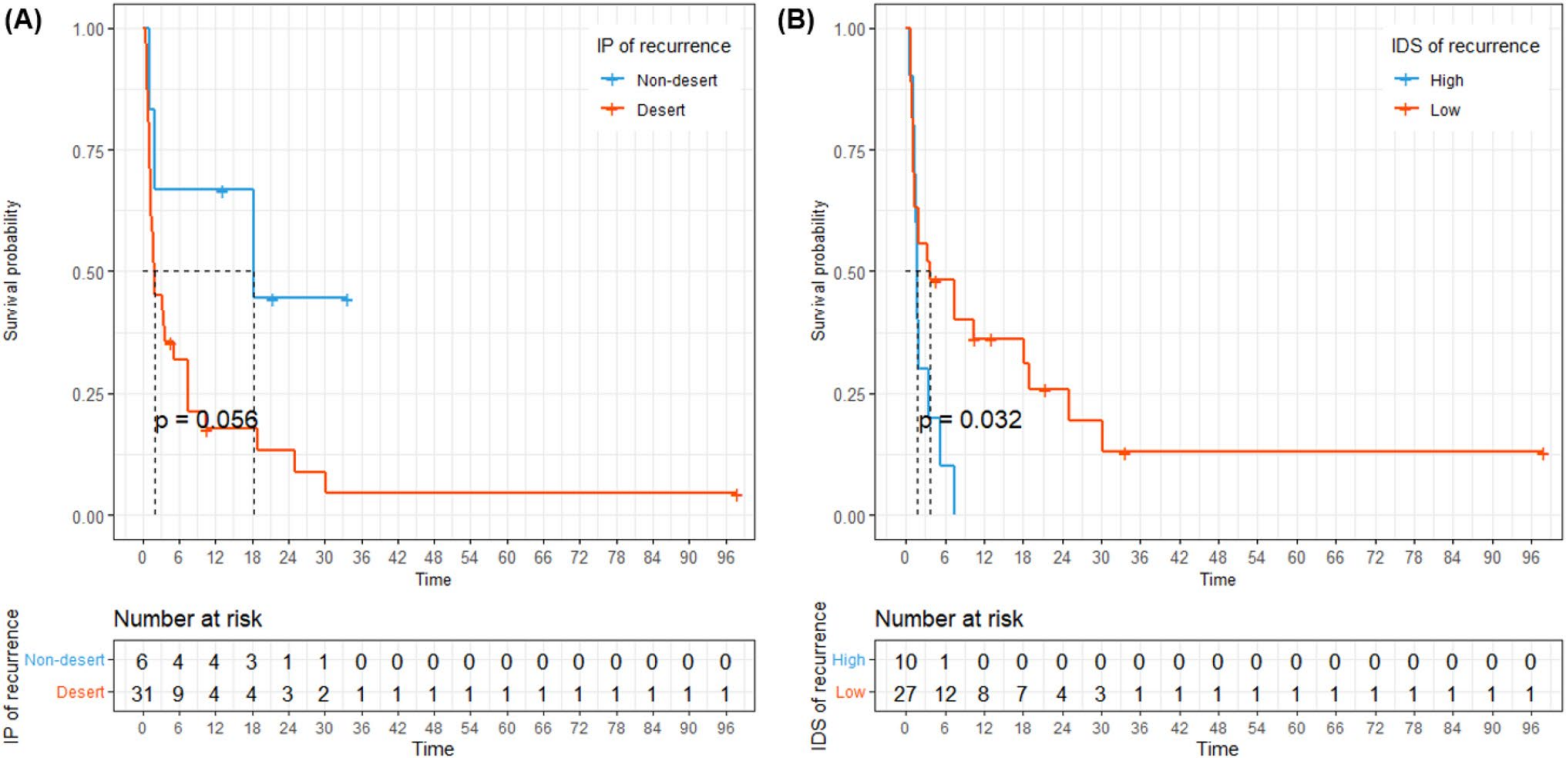
Progression-free survival (PFS) based on the tumor microenvironment of recurrent tumor. **A** PFS based on the immune phenotype (desert vs. non-desert); **B** PFS based on the immune-desert score

To clarify whether IP is also a predictive biomarker of ICI treatment, we categorized IP in both the initial tumor and recurrence into the desert and non-desert groups, and analyzed the corresponding outcomes of ICI treatment. In the case of the initial tumor, there was no difference in the PFS between the desert and non-desert groups (Supplementary Fig. 3, median 7.5 vs. 1.7 months, *P* = 0.42). For IP of recurrent tumor, the desert group exhibited numerically shorter PFS than the non-desert group, although statistically insignificant (Fig. 4A, median 1.9 vs. 18.2 months, *P* = 0.056).

We conducted a comparative analysis of survival outcomes based on IDS levels in recurrent tumor to confirm if the IP of recurrence was associated with survival outcomes related to ICI treatment. The optimal cut-off level for determining high and low IDS was 92.77. Using this threshold, 10 patients (27%) were in the high-IDS group and the remaining 27 (73%) were in the low-IDS group. Patients with a higher IDS (> 92.77%) exhibited shorter PFS (Fig. 4B, median 1.7 vs. 3.7 months, *P* = 0.032).

## Discussion

In this study, we compared the TIL density and IP between the initial tumor and recurrence to assess whether there was a difference in the TME of R/M HNSCC. Both iTIL and sTIL densities were significantly lower in the recurrent tumor compared to the initial tumor. Regarding spatial distribution, the IS was lower, and the IDS was higher for recurrences. As a result, the recurrent tumor demonstrated a higher proportion of immune-desert IP. The change in sTIL density was a predictive biomarker for the response to ICI and survival outcomes. IP in the initial tumor was not associated with PFS, whereas high IDS at recurrence was associated with shorter PFS.

With the advent of immunotherapy, various types of ICIs have been widely used for the treatment of R/M HNSCC [21]. However, owing to an insufficient overall response rate to ICIs and the risk of hyperprogressive disease, there is an urgent need to find predictive biomarkers [22]. Studies on the prognostic value of TILs in HNSCC have been documented [10, 23]; however, research on their association with ICI use is limited. Moreover, these studies utilized tissues from initial tumors, and the changes between initial tumors and recurrence or their relevance to ICI in HNSCC have not been well elucidated. Numerous studies have compared the TME of primary tumors and metastases for PD-L1 expression, albeit with conflicting results. Several reports have described concordance between primary tumors and metastases with respect to PD-L1 expression in HNSCC [24, 25]. In contrast, several studies have indicated significant discordance between primary tumors and metastatic lesions [26, 27]. From the perspective of TIL, a report documents no significant difference between primary and metastatic lesions in NSCLC [15], whereas in various other cancer types, there are reports show decreased TILs in recurrence [28–30]. Although rare in the context of HNSCC, one study compared the CD8 + TIL levels between the initial tumor and regional recurrence [31]. Our data are consistent with those of the previous study, indicating a decrease in CD8 + TIL levels during recurrence and its association with poor prognosis. In contrast, Chang et al*.* reported no differences in PD-L1, PD-1, and CD8 + TIL between primary and recurrent tumors [11]. However, the analysis involved a small sample size of seven patients only. Our study, which leverages AI for enhanced precision, is likely to validate these differences.

In our study, both iTIL and sTIL significantly decreased during recurrence, leading to a substantial increase in the proportion of immune-desert IP. Interestingly, 92% of patients who were initially having a non-desert IP transitioned to immune-desert IP in recurrence. This is the first study to analyze the differences in the TME in TIL density and IP between the initial and recurrent HNSCC. The low TIL density and a high proportion of desert IP in recurrence indicate a significant contribution of weakened anti-tumor immunity to tumor progression.

Recurrence often exhibits a decreasing trend in TIL density, but this varies among patients; intriguingly, some show an increase during recurrence, albeit at low rates, i.e., 38% and 19% for iTIL and sTIL, respectively. Studies analyzing changes in TIL in HNSCC have reported that prior radiotherapy contributes to a decrease in TIL density [12, 31]. Ock et al*.* reported an association between chemotherapy and changes in PD-L1 expression in HNSCC [13], suggesting that the TME of HNSCC is significantly affected by previous treatments. Our data showed a tendency for younger age in the group with decreased iTIL density, suggesting a potential effect of more aggressive and extensive treatment in this group of patients. Interestingly, the time interval between specimens did not affect changes in TILs. This finding contrasts with research on soft tissue sarcomas, indicating an association between changes in CD8 + TIL and PD-L1 + lymphocytes and the time interval between tissue collections [29]. Considering these factors, performing a re-biopsy before ICI treatment in HNSCC may be beneficial, even if the time to recurrence is short. This approach could provide a more accurate evaluation of the TME and potentially aid in predicting treatment response.

The present study demonstrated the predictive value of TIL and IP analyzed using Lunit SCOPE IO, an AI-powered spatial TIL analyzer, as biomarkers for ICI efficacy in patients with recurrent HNSCC. TILs analyzed using Lunit SCOPE IO are advantageous for simple and convenient evaluation, requiring only H&E-stained slides. In contrast, in other solid tumor types, differentiating TILs into specific T-cell subsets through immunohistochemistry can be a more effective biomarker than total TIL. In particular, there is extensive research on CD4 + and CD8 + TIL because each represents a T-cell subset directly associated with anti-tumor immunity, functioning as helper cells and cytotoxic T cells, respectively [10]. However, in HNSCC, T-cell subsets are significantly affected by the primary tumor location, and results on its prognostic value are inconsistent [32]. Moreover, Ward et al*.* reported that the predictive power of TILs based on the analysis of T-cell subsets through immunohistochemistry was not superior to that of total TIL and found that TIL based on simple H&E-stained images had the best predictive accuracy [33]. We considered the spatial distribution of TILs, distinguished it between the intratumoral and stromal areas, and calculated the TIL density in each area. Unlike changes in iTIL density, those in sTIL density were associated with response to ICIs and survival outcomes, underscoring the potential importance of changes in sTIL density. Several studies have emphasized the utility of sTIL over iTIL as biomarkers for various cancer types, including head and neck cancers [34–36]. There are several possible explanations for this. The immune response at the tumor margin is crucial for invasion toward the periphery for progression, and sTIL infiltration is associated with vascular and lymphatic invasion at the invasive margin [37, 38]. In addition, our data showed that iTIL density was significantly lower than sTIL density in both initial tumors and recurrences, and the magnitude of the changes was also low. This difference could make sTIL a more reliable factor, potentially affecting these results.

We analyzed the changes in TIL density and the effect of spatial distribution on the use of ICIs. Although there was no statistical significance, desert IP in recurrence showed a shorter PFS than non-desert IP, unlike in the initial tumor, where there was no difference between IPs. For recurrence, the predominance of desert IP and insufficient number of non-desert IP cases may contribute to the lack of statistically significance. Considering this possibility, we analyzed survival outcomes using IDS, a spatial TIL infiltration sparsity measure. The results showed that a high IDS for recurrence was associated with shorter PFS. While previous studies have demonstrated the overall prognostic value of the TME in the initial tumor tissues, our results suggest that tissues after recurrence may be more suitable as predictors of survival outcomes from the point of ICI use than the initial tissues. Our study highlights the importance of identifying appropriate tissue biomarkers for ICI use in recurrent HNSCC.

This study has some limitations that warrant further consideration. The first was the small sample size analyzed. A heterogeneous group of patients with various primary tumor sites was included, and the impact of tumor sites on the TME could not be assessed due to the small sample size. Second, we were unable to analyze the impact of PD-L1 expression in the TME due to the lack of PD-L1 data. Although several studies have indicated that TILs are more critical biomarkers than PD-L1 expression or its change [31, 39], information on changes in both PD-L1 and TILs in recurrent tumors can provide broader insights into the TME. Additionally, considering PD-L1 and TILs together for patient stratification may have a better predictive value than classification based solely on TILs [40]. Third, Lunit SCOPE IO operates based on simple H&E-stained WSIs and does not offer additional data on the cellular characterization or functional activity of TILs. However, on the other hand, it has the advantage of being immediately applicable without additional staining and can be applied to multiple cases.

Despite these limitations, our study is the first to compare and analyze TIL density and IP through paired analysis of tissues from both initial and recurrent HNSCC. The present study demonstrated that the density and spatial distribution of TILs analyzed using Lunit SCOPE IO, an AI-powered TIL analyzer, are useful biomarkers for optimizing ICI use for R/M HNSCC. Further research with a larger series is warranted to validate the changes in the TME and its predictive value for ICI treatments in HNSCC.

## Conclusion

In conclusion, TIL density decreases during recurrence compared to the initial stage, ultimately resulting in a frequent transition to immune-desert IP. Patients with decreased sTIL density during recurrence, compared with the initial tumor, exhibited a poor response to ICI and unfavorable survival outcomes. The spatial TIL distribution in recurrence demonstrated a better predictive value for survival outcomes than that in the initial tumor; high IDS in recurrence was also associated with shorter PFS. Therefore, re-performing tissue analysis after recurrence could provide a more accurate assessment of the TME and serve as a biomarker for using ICI in R/M HNSCC.

## Supplementary Information

Supplementary Material 1

## Data Availability

The datasets generated during the current study are available from the corresponding author on reasonable request.

## References

[1] Johnson DE, et al. Head and neck squamous cell carcinoma. Nat Rev Dis Primers. 2020;6(1):92. 33243986 10.1038/s41572-020-00224-3 PMC7944998

[2] Burtness B, et al. Pembrolizumab alone or with chemotherapy versus cetuximab with chemotherapy for recurrent or metastatic squamous cell carcinoma of the head and neck (KEYNOTE-048): a randomised, open-label, phase 3 study. Lancet. 2019;394(10212):1915–28. 31679945 10.1016/S0140-6736(19)32591-7

[3] Harrington KJ, et al. Nivolumab versus standard, single-agent therapy of investigator’s choice in recurrent or metastatic squamous cell carcinoma of the head and neck (CheckMate 141): health-related quality-of-life results from a randomised, phase 3 trial. Lancet Oncol. 2017;18(8):1104–15. 28651929 10.1016/S1470-2045(17)30421-7 PMC6461049

[4] Ferris R, et al. Durvalumab with or without tremelimumab in patients with recurrent or metastatic head and neck squamous cell carcinoma: EAGLE, a randomized, open-label phase III study. Ann Oncol. 2020;31(7):942–50. 32294530 10.1016/j.annonc.2020.04.001

[5] Guigay J, et al. Avelumab for platinum-ineligible/refractory recurrent and/or metastatic squamous cell carcinoma of the head and neck: phase Ib results from the JAVELIN Solid Tumor trial. J ImmunoTherapy Cancer. 2021. 10.1136/jitc-2021-002998. 10.1136/jitc-2021-002998 PMC8524383 34663640

[6] Zech HB, et al. Phase III study of nivolumab alone or combined with ipilimumab as immunotherapy versus standard of care in resectable head and neck squamous cell carcinoma. Future Oncol. 2020;16(36):3035–43. 32902312 10.2217/fon-2020-0595

[7] Oliva M, et al. Immune biomarkers of response to immune-checkpoint inhibitors in head and neck squamous cell carcinoma. Ann Oncol. 2019;30(1):57–67. 30462163 10.1093/annonc/mdy507 PMC6336003

[8] Park JC, Krishnakumar HN, Saladi SV. Current and future biomarkers for immune checkpoint inhibitors in head and neck squamous cell carcinoma. Curr Oncol. 2022;29(6):4185–98. 35735443 10.3390/curroncol29060334 PMC9221564

[9] Li F, et al. The association between CD8+ tumor-infiltrating lymphocytes and the clinical outcome of cancer immunotherapy: a systematic review and meta-analysis. EClinicalMedicine. 2021. 10.1016/j.eclinm.2021.101134. 34585125 10.1016/j.eclinm.2021.101134 PMC8452798

[10] de Ruiter EJ, et al. The prognostic role of tumor infiltrating T-lymphocytes in squamous cell carcinoma of the head and neck: a systematic review and meta-analysis. Oncoimmunology. 2017;6(11): e1356148. 29147608 10.1080/2162402X.2017.1356148 PMC5674970

[11] Chang H, et al. Overexpression of PD-L2 is associated with shorter relapse-free survival in patients with malignant salivary gland tumors. OncoTargets Therapy. 2017;10:2983–92. 28652780 10.2147/OTT.S134589 PMC5476710

[12] Pflumio C, et al. Expression of immune response biomarkers (PD-L1, p16, CD3+ and CD8+ TILs) in recurrent head and neck squamous cell carcinoma within previously irradiated areas. Oncol Rep. 2021;45(3):1273–83. 33432367 10.3892/or.2021.7928

[13] Ock C-Y, et al. Changes in programmed death-ligand 1 expression during cisplatin treatment in patients with head and neck squamous cell carcinoma. Oncotarget. 2017;8(58):97920. 29228662 10.18632/oncotarget.18542 PMC5716702

[14] Jung HA, et al. A phase II study of nivolumab plus gemcitabine in patients with recurrent or metastatic nasopharyngeal carcinoma (KCSG HN17–11). Clin Cancer Res. 2022;28(19):4240–7. 35819451 10.1158/1078-0432.CCR-22-1238

[15] Park S, et al. Artificial intelligence-powered spatial analysis of tumor-infiltrating lymphocytes as complementary biomarker for immune checkpoint inhibition in non–small-cell lung cancer. J Clin Oncol. 2022;40(17):1916. 35271299 10.1200/JCO.21.02010 PMC9177249

[16] Kim DH, et al. Artificial intelligence-powered spatial analysis of tumor-infiltrating lymphocytes as a predictive biomarker for axitinib in adenoid cystic carcinoma. Head Neck. 2023;45(12):3086–95. 37828867 10.1002/hed.27537

[17] Kim DH, et al. Artificial intelligence-powered spatial analysis of tumor-infiltrating lymphocytes as a biomarker in locally advanced unresectable thymic epithelial neoplasm: a single-center, retrospective, longitudinal cohort study. Thorac Cancer. 2023;14(30):3001–11. 37675597 10.1111/1759-7714.15089 PMC10599973

[18] Kim DH, et al. Comparison of tumor microenvironments between primary tumors and lymph node metastases in head and neck squamous cell carcinoma and their predictive role in immune checkpoint inhibitor treatment. Cells. 2024;13(18):1557. 39329741 10.3390/cells13181557 PMC11429639

[19] Ayers M, et al. IFN-γ-related mRNA profile predicts clinical response to PD-1 blockade. J Clin Investig. 2017;127(8):2930–40. 28650338 10.1172/JCI91190 PMC5531419

[20] Chen DS, Mellman I. Elements of cancer immunity and the cancer-immune set point. Nature. 2017;541(7637):321–30. 28102259 10.1038/nature21349

[21] Poulose JV, Kainickal CT. Immune checkpoint inhibitors in head and neck squamous cell carcinoma: a systematic review of phase-3 clinical trials. World J Clin Oncol. 2022;13(5):388. 35662989 10.5306/wjco.v13.i5.388 PMC9153072

[22] Park JH, et al. Hyperprogressive disease and its clinical impact in patients with recurrent and/or metastatic head and neck squamous cell carcinoma treated with immune-checkpoint inhibitors: Korean cancer study group HN 18–12. J Cancer Res Clin Oncol. 2020;146:3359–69. 32671504 10.1007/s00432-020-03316-5 PMC11804517

[23] Spector ME, et al. Prognostic value of tumor-infiltrating lymphocytes in head and neck squamous cell carcinoma. JAMA Otolaryngol Head Neck Surg. 2019;145(11):1012–9. 31486841 10.1001/jamaoto.2019.2427 PMC6735419

[24] Chen T-C, et al. Associations among pretreatment tumor necrosis and the expression of HIF-1α and PD-L1 in advanced oral squamous cell carcinoma and the prognostic impact thereof. Oral Oncol. 2015;51(11):1004–10. 26365985 10.1016/j.oraloncology.2015.08.011

[25] Kaur A, Jamshidi P, Paintal A. PD-L1 combined positive score (CPS) scoring in p16+ oropharyngeal squamous cell carcinoma (OPSCC): a comparison of scoring in paired primary tumors and lymph node metastases. in Laboratory investigation. 2020. Nature Publishing Group, New York, NY, USA.

[26] Scognamiglio T, Chen Y-T. Beyond the percentages of PD-L1-positive tumor cells: induced versus constitutive PD-L1 expression in primary and metastatic head and neck squamous cell carcinoma. Head Neck Pathol. 2018;12:221–9. 28948509 10.1007/s12105-017-0857-3 PMC5953879

[27] Straub M, et al. CD274/PD-L1 gene amplification and PD-L1 protein expression are common events in squamous cell carcinoma of the oral cavity. Oncotarget. 2016;7(11):12024. 26918453 10.18632/oncotarget.7593 PMC4914266

[28] Ogiya R, et al. Comparison of tumor-infiltrating lymphocytes between primary and metastatic tumors in breast cancer patients. Cancer Sci. 2016;107(12):1730–5. 27727484 10.1111/cas.13101 PMC5198965

[29] Zheng B, et al. Changes in the tumor immune microenvironment in resected recurrent soft tissue sarcomas. Ann Transl Med. 2019;7(16):387. 31555701 10.21037/atm.2019.07.43 PMC6736819

[30] Predina J, et al. Changes in the local tumor microenvironment in recurrent cancers may explain the failure of vaccines after surgery. Proc Natl Acad Sci. 2013;110(5):E415–24. 23271806 10.1073/pnas.1211850110 PMC3562776

[31] So YK, et al. An increase of CD8+ T cell infiltration following recurrence is a good prognosticator in HNSCC. Sci Rep. 2020;10(1):20059. 33208791 10.1038/s41598-020-77036-8 PMC7674485

[32] Borsetto D, et al. Prognostic significance of CD4+ and CD8+ tumor-infiltrating lymphocytes in head and neck squamous cell carcinoma: a meta-analysis. Cancers. 2021;13(4):781. 33668519 10.3390/cancers13040781 PMC7918220

[33] Ward M, et al. Tumour-infiltrating lymphocytes predict for outcome in HPV-positive oropharyngeal cancer. Br J Cancer. 2014;110(2):489–500. 24169344 10.1038/bjc.2013.639 PMC3899750

[34] Oguejiofor K, et al. Stromal infiltration of CD8 T cells is associated with improved clinical outcome in HPV-positive oropharyngeal squamous carcinoma. Br J Cancer. 2015;113(6):886–93. 26313665 10.1038/bjc.2015.277 PMC4578081

[35] Kim DH, et al. Artificial intelligence-powered spatial analysis of tumor-infiltrating lymphocytes as a predictive biomarker for axitinib in adenoid cystic carcinoma. Head Neck. 2023. 10.1002/hed.27537. 37828867 10.1002/hed.27537

[36] Pagès F, et al. In situ cytotoxic and memory T cells predict outcome in patients with early-stage colorectal cancer. J Clin Oncol. 2009;27(35):5944–51. 19858404 10.1200/JCO.2008.19.6147

[37] Sun X-F, Zhang H. Clinicopathological significance of stromal variables: angiogenesis, lymphangiogenesis, inflammatory infiltration, MMP and PINCH in colorectal carcinomas. Mol Cancer. 2006;5:1–20. 17026740 10.1186/1476-4598-5-43 PMC1618857

[38] Al-Shibli KI, et al. Prognostic effect of epithelial and stromal lymphocyte infiltration in non–small cell lung cancer. Clin Cancer Res. 2008;14(16):5220–7. 18698040 10.1158/1078-0432.CCR-08-0133

[39] Tumeh PC, et al. PD-1 blockade induces responses by inhibiting adaptive immune resistance. Nature. 2014;515(7528):568–71. 25428505 10.1038/nature13954 PMC4246418

[40] Kuba K, et al. A retrospective analysis of tumor infiltrating lymphocytes in head and neck squamous cell carcinoma patients treated with nivolumab. Sci Rep. 2022;12(1):22557. 36581686 10.1038/s41598-022-27237-0 PMC9800384

