# Supplementary material for: Changes in the tumor microenvironment in recurrent head and neck squamous cell carcinoma and its implication on efficacy of immune checkpoint inhibitors
Source: Discov Oncol. 2024 Nov 20;15:686. doi: 10.1007/s12672-024-01504-0 (PMC11579274; doi:10.1007/s12672-024-01504-0)
Supplement: Supplementary file 1 — Supplementary Material 1 [file 12672_2024_1504_MOESM1_ESM.docx]

**Supplemental Appendix**

**Changes in the tumor microenvironment in recurrent head and neck squamous cell carcinoma and its implication on efficacy of immune checkpoint inhibitors**

Dong Hyun Kim, Mingu Kang, Gahee Park, Yoojoo Lim, Chan-Young Ock, Jiwon Koh, Yoon Kyung Jeon, Kyeong Cheon Jung, Soon-Hyun Ahn, Eun-Jae Chung, Seong-Keun Kwon, and Bhumsuk Keam

**Correspondence to:**

Bhumsuk Keam, MD, PhD

Department of Internal Medicine, Seoul National University Hospital, 101, Daehak-ro, Jongro-gu, Seoul 03080, Republic of Korea

Tel +82-2-2072-7215

E-mail [bhumsuk@snu.ac.kr](mailto:bhumsuk@snu.ac.kr)

**Supplemental Figure**

**Fig. S1. Correlation of TIL density and immune score between initial and recurrent tumor.**

**
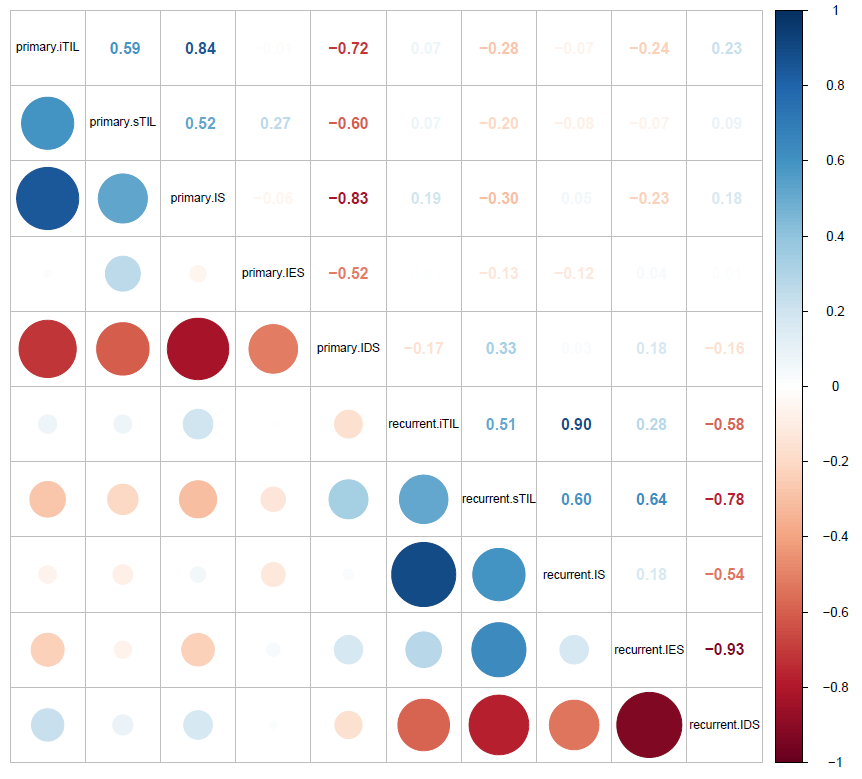
**

The upper half of the diagonal represents correlation coefficients, with darker font colors indicating strong correlations. The bottom half displays them through the size and color of circles.

Abbreviations: TIL, tumor-infiltrating lymphocyte; iTIL, intratumoral TIL; sTIL, stromal TIL; IS, inflamed score; IES, immune-excluded score; IDS, immune-desert score.

**Fig. S2. Survival outcomes based on changes in intratumoral TIL density. (A) Progression-free survival; (B) Overall survival.**


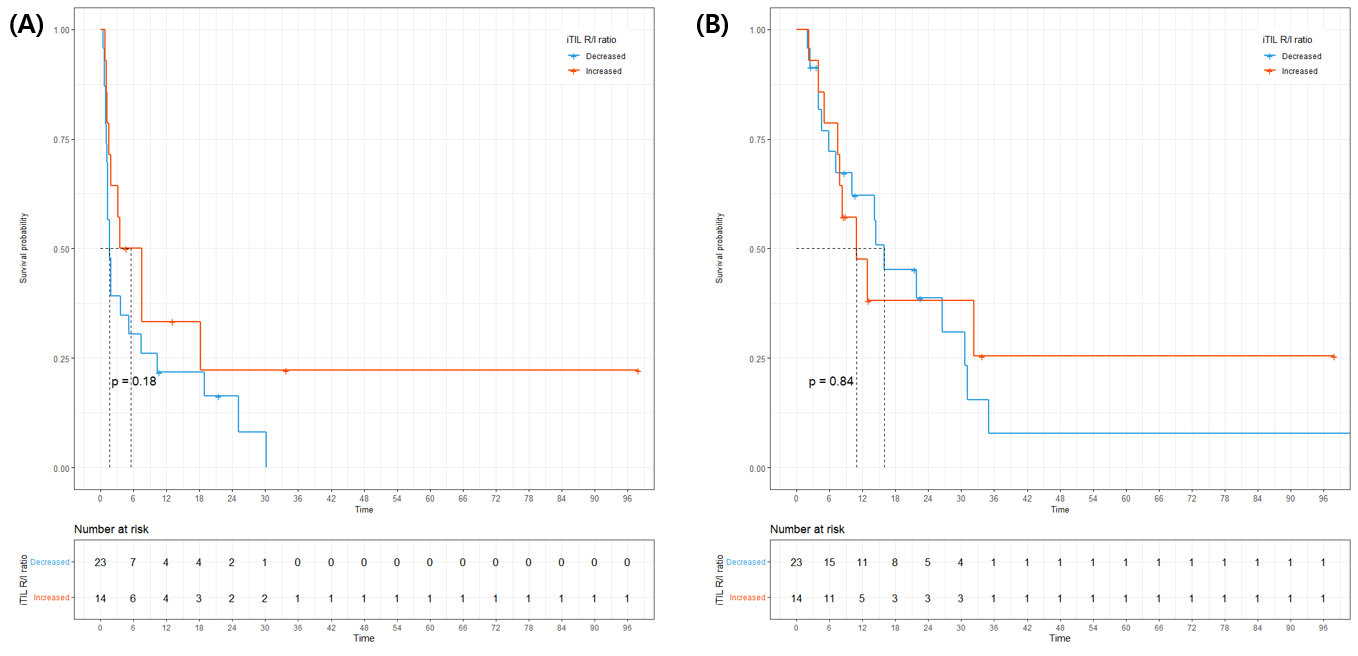


**Fig. S3. Progression-free survival based on the immune phenotype of the initial tumor.**


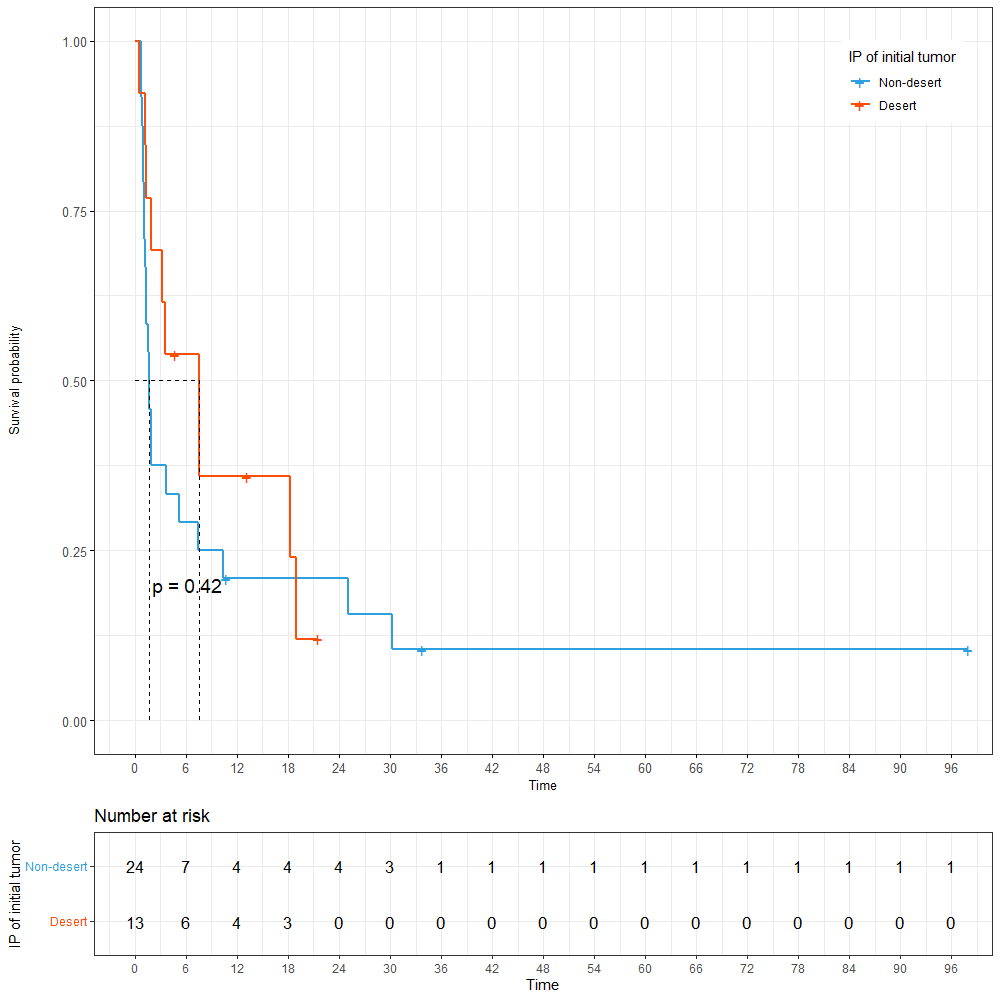


**Supplemental Table**

**Table S1. Detailed information on recurrent tumor tissue.**

|  | **N = 37** |
| --- | --- |
| **Tissue acquisition method, n (%)**  Needle biopsy  Excisional biopsy/surgery | 30 (81.1)  7 (18.9) |
| **Site, n (%)**  Primary site  Cervical lymph node metastasis  Distant metastasis  Lung/pleura  Bronchus  Skin/soft tissue | 14 (37.8)  11 (29.7)  12 (32.4)  8  2  2 |

**Table S2. Univariate and multivariate logistic regression analysis for overall response.**

|  | **Univariable** | | **Multivariable** | |
| --- | --- | --- | --- | --- |
|  | **OR (95% CI)** | ***P*-value** | **OR (95% CI)** | ***P*-value** |
| **Age > 60** | 5.14 (1.17-22.69) | 0.031 | 4.97 (0.99-24.9) | 0.051 |
|  |  |  |  |  |
| **Current or former smoker** | 1.84 (0.43-7.77) | 0.409 |  |  |
|  |  |  |  |  |
| **Initial stage 4** | 2 (0.43-9.26) | 0.375 |  |  |
|  |  |  |  |  |
| **Primary tumor site, oral cavity** | 0.77 (0.19-3.11) | 0.718 |  |  |
|  |  |  |  |  |
| **Differentiation, well** | 0.23 (0.03-2.16) | 0.201 |  |  |
|  |  |  |  |  |
| **Number of previous lines of chemotherapy**  0-1  ≥ 2 | 1.00  2.17 (0.52-9.09) | 0.291 |  |  |
|  |  |  |  |  |
| **ICI treatment**  PD-1 inhibitor  PD-L1 inhibitor  PD-1 or PD-L1 inhibitor + CTLA-4  inhibitor | 1.00  0.43 (0.04-4.35)  0.43 (0.04-4.35) | 0.471  0.471 |  |  |
|  |  |  |  |  |
| **Increased intratumoral TIL** | 2.12 (0.52-8.7) | 0.295 |  |  |
| **Increased stromal TIL** | 8.21 (1.3-51.99) | 0.025 | 7.9 (1.09-57.25) | 0.041 |

OR, odds ratio; CI, confidence interval; ICI, immune checkpoint inhibitor; PD-1, programmed cell death-1; PD-L1, programmed cell death-ligand 1; CTLA-4, cytotoxic T-lymphocyte-associated protein 4; TIL, tumor-infiltrating lymphocyte.

**Table S3. Univariate Cox proportional hazard model for PFS and OS.**

|  | **Progression-free survival** | | **Overall survival** | |
| --- | --- | --- | --- | --- |
|  | **HR (95% CI)** | ***P*-value** | **HR (95% CI)** | ***P*-value** |
| **Age > 60** | 0.6 (0.28-1.29) | 0.190 | 0.58 (0.24-1.41) | 0.227 |
|  |  |  |  |  |
| **Current or former smoker** | 0.66 (0.3-1.44) | 0.297 | 0.89 (0.37-2.15) | 0.802 |
|  |  |  |  |  |
| **Initial stage 4** | 0.52 (0.25-1.09) | 0.084 | 0.79 (0.35-1.82) | 0.587 |
|  |  |  |  |  |
| **Primary tumor site, oral cavity** | 1.74 (0.85-3.54) | 0.128 | 0.98 (0.44-2.19) | 0.961 |
|  |  |  |  |  |
| **Differentiation, well** | 2.76 (1.19-6.39) | 0.018 | 2.11 (0.82-5.41) | 0.120 |
|  |  |  |  |  |
| **Number of previous lines of chemotherapy**  0-1  ≥ 2 | 1.00  0.66 (0.3-1.44) | 0.245 | 1.00  1.31 (0.57-2.98) | 0.523 |
|  |  |  |  |  |
| **ICI treatment**  PD-1 inhibitor  PD-L1 inhibitor  PD-1 or PD-L1 inhibitor + CTLA-4  inhibitor | 1.00  0.71 (0.24-2.06)  0.78 (0.27-2.29) | 0.527  0.655 | 1.00  0.82 (0.27-2.46)  1.48 (0.53-4.1) | 0.718  0.451 |
|  |  |  |  |  |
| **Increased intratumoral TIL** | 0.6 (0.28-1.28) | 0.184 | 0.92 (0.4-2.1) | 0.835 |
| **Increased stromal TIL** | 0.13 (0.03-0.57) | 0.007 | 0.24 (0.06-1.01) | 0.052 |

HR, hazard ratio; CI, confidence interval; ICI, immune checkpoint inhibitor; PD-1, programmed cell death-1; PD-L1, programmed cell death-ligand 1; CTLA-4, cytotoxic T-lymphocyte-associated protein 4; TIL, tumor-infiltrating lymphocyte.
